# A cluster-randomized trial of a complex intervention to encourage deprescribing antidepressants in nursing home residents with dementia: a study protocol

**DOI:** 10.1186/s13063-022-06368-9

**Published:** 2022-05-16

**Authors:** Pernille Hølmkjær, Anne Holm, Gritt Overbeck, Maarten Pieter Rozing

**Affiliations:** Department of Public Health, Section of General Practice and Research Unit for General Practice, University of Copenhagen, Copenhagen, Denmark

**Keywords:** Dementia, Deprescribing, Antidepressants, BPSD

## Abstract

**Background:**

The effectiveness of psychotropic medication on behavioral and psychological symptoms of dementia (BPSD) is limited, while associated with a higher risk of adverse events. Non-pharmacological treatment of BPSD is advocated as treatment of first choice. However, many general practitioners (GPs) find it difficult to initiate deprescribing, and when attempting to discontinue psychotropic medication in nursing home residents, they face many barriers. Therefore, we hypothesize that an intervention aimed at improving communication with and involvement of nursing home staff, relatives, and patients by GPs can optimize the pharmacological treatment of BPSD. The aim is to reduce the use of antidepressants in nursing home residents with dementia without increasing morbidity or mortality.

**Objective:**

The primary outcome is reduction of antidepressant. Secondary outcomes include difference in use of other psychotropic medication, mortality, morbidity, and severity of BPSD.

**Method:**

The study is a cluster-randomized controlled trial based in general practices in Denmark. We aim to include 22 practices, each of which will recruit up to 15 patients with dementia living in nursing homes. The intervention period is 3 months, and the total study period is 1 year. Randomization is 1:1 to intervention and control group by computer algorithm. Both groups receive education on BPSD and its evidence-based treatment. The intervention includes three tailored components; (1) teaching material and training to be used by the GP to educate nursing home staff on BPSD, (2) a pre-visit reflection tool to encourage nursing home staff to evaluate symptoms and reflect on relatives involvement in the discontinuation process; and (3) a dialog tool to facilitate shared decision making on optimization of BPSD treatment during the visits at the nursing home. The control group includes enhanced care as usual. The primary and secondary outcomes will be assessed at the end of the study period. A process evaluation will be conducted to assess the implementability.

**Discussion:**

We anticipate that the intervention will optimize the treatment of BPSD with antidepressants for nursing homes residents and enhance compliance with reduction of medication. The process evaluation should provide insights into the barriers and facilitators to changing the current practice of deprescribing.

**Trial registration:**

Clinicaltrials.gov NCT04985305. Registered on 30 July 2021.

**Supplementary Information:**

The online version contains supplementary material available at 10.1186/s13063-022-06368-9.

## Introduction

It is estimated that more than 87,000 in Denmark are living with dementia, with more than 8000 new cases each year. The majority of older people with dementia are living at home but in a minority, problems with daily activities necessitate relocation to a nursing home [1]. Besides cognitive impairment, up to 90 % of the institutionalized older people with dementia experience behavioral and psychological symptoms of dementia (BPSD) such as anxiety, agitation, hallucinations, depression, and apathy [2]. An overuse of antidepressants is reported, and about half of all nursing home residents in Denmark receive at least one antidepressant as well as other psychotropic medication such as antipsychotics, anxiolytics, and hypnotics in addition to the antidepressants [3–7]. However, recent research has shown that antidepressants have limited benefits in older people suffering from dementia and that they can inflict harm such as increasing the risk of falls and cardiovascular adverse events in institutionalized older persons [8–10]. Given the limited effectiveness of psychotropic medication and its considerable risk of side-effects such as dizziness and falls, the use of antipsychotics and anxiolytics have been recommended against for a long period, while recommendations considering antidepressants have been conflicting [11–16]. Danish national guidelines recommend against the use of antidepressants in older people suffering from dementia and advocate non-pharmacological treatment of BPSD as treatment of first choice [17].

Despite the Danish guidelines, a recent study showed that moving into a nursing home was accompanied by an increase in the number of new drug treatments including antidepressants, and that this number remained unchanged for at least two years [4]. Studies on the implementation and retention of strategies to discontinue psychotropic medication have shown varying effects [18–20]. A recent qualitative systematic review shows that discontinuation is often hindered by several factors. Firstly, the general practitioner (GP) does not get the necessary information from the staff, secondly, both relatives and staff can have concerns about the reduction or discontinuation of psychotropic medication, and thirdly, the GP does not feel sufficiently competent to make medication adjustments in this patient group [21]. These factors complicate the evaluation and adjustment of pharmacological treatment of neuropsychiatric symptoms. A Danish national strategy to try to reduce antipsychotics has been initialized in 2020, but there is no focused initiative to reduce antidepressants [22].

In light of these considerations, we have developed an intervention specifically aimed at improving communication and collaboration between GPs, nursing home staff, relatives, and patients to optimize the pharmacological treatment of BPSD. The intervention is developed through a tailoring process which is described elsewhere and not yet published. The tailoring process ensures the involvement of GPs and adjustment of the intervention to GPs. We have not planned any individual tailoring. The process evaluation will investigate if GPs tailor individual elements such as the teaching of nursing home staff on their own. This complex intervention purports to (1) increase the knowledge of neuropsychiatric symptoms in dementia for the nursing home staff and (2) encourage the involvement of staff and relatives in the deprescription process by employing a pre-visit reflection tool and a dialog tool [23].

This study protocol aims to describe the evaluation of the effectiveness and feasibility of this complex intervention by improving the deprescription process of antidepressants and other psychotropic medication in institutionalized older people by the general practitioners.

### Trial design

This trial is designed as a cluster-randomized, non-blinded parallel-group superiority trial with a 1:1 allocation ratio. Enhanced care-as-usual will be used as a comparator as this constitutes the most naturalistic approach and will increase the feasibility. A process evaluation will be undertaken during the study period.

## Methods / design

This study is reported using the SPIRIT reporting guidelines [24].

### Trial setting

The study takes place in the Capital Region of Denmark. Earlier research has shown that this region has the highest prescription rates of psychotropic medication for older persons with dementia in Denmark, and an intervention is expected to have the highest impact here [25]. In Denmark, the healthcare system is free of charge for everyone with a social security number. The GP functions as a gatekeeper to the secondary healthcare system, referring when necessary to hospitals and most office-based specialists. Furthermore, the GP represents the primary contact with the nursing homes in the municipalities concerning the patients residing in such facilities.

Most of the patients, when moving into a nursing home, are attended by a primary attending GP affiliated with a nursing home (hereafter called nursing home physician), but some continue with their regular GP.

In the national collective agreement for GPs in Denmark, GPs may conduct a yearly health check on older patients aged ≥75, with a higher reimbursement than a regular consultation. This health check is not mandatory and can be planned and held as GPs see fit. It is recommended to include the following [26, 27]

- To gain knowledge of the resources and functionality
- To identify and, if possible, prevent and limit health care challenges
- To evaluate and, if possible, revise the patients’ medical chart
- To collect knowledge of the older person’s daily living conditions, so the GP is well equipped to be a competent partner in interdisciplinary health care work.

In the Capital Region, there are 187 nursing homes and 125 have a regular nursing home physician. The nursing homes encompass both private and community-owned, with only a few solely for residents with dementia [28]. Nursing homes vary in size from 10 to 193 residents. The staff works 8-h shifts and includes nurses, nurses’ assistants, health care personnel, and often physiotherapists and ergotherapists. As all municipalities are organized differently, the number of staff and their education level varies greatly [29]. Around half of Danish nursing home residents either have a diagnosis of dementia or experience cognitive deficiencies to some degree without a confirmed dementia diagnosis [30, 31].

### Identification, eligibility assessment, and recruitment

#### In- and exclusion criteria for GPs

GPs are eligible for participation if they are employed in the Capital Region and are nursing home physicians for at least ten patients. If not enough nursing home physicians are available for inclusion, GPs with no direct affiliation, but who have patients residing in nursing homes, can be included. GPs that are currently participating in trials similar to ours are excluded.

#### Identification and recruitment of GPs

There is no national registry for nursing home physicians in Denmark. A national registry for nursing homes exists, and a list of all nursing homes in the Capital Region is compiled including a contact email for the nursing home. First, an email is sent to all nursing homes on the list requesting the name of their nursing home physician. If no answer is received, the research team calls the nursing home to ask for the name of the nursing home physician. A list of all nursing home physicians is then compiled. Afterwards, an invitation is sent out to all nursing home physicians and will be posted in a closed group for nursing home physicians on Facebook and a newsletter sent to all GPs in the Capital Region of Denmark.

A reminder will be sent two months after, followed by direct contact to the nursing home physician by telephone. If less than 22 nursing home physicians have expressed positive interest in participating, another round of letters will be sent, inviting GPs not affiliated with a nursing home (hereafter called non-nursing home physicians) to take part as well. We aim to include at least 22 GPs.

After completion of GP recruitment, GPs will receive written instructions on how to identify eligible patients. All nursing homes attended by the GP will be included.

All GPs participating in the study will receive reimbursement for administrative tasks in connection with the project.

#### In- and exclusion criteria for patients

Patients are eligible for participation if they are living in a nursing home that participates in the trial. Furthermore, they must fulfill the following inclusion criteria:

- ≥72 years old
- A diagnosis of dementia or severe cognitive impairment as judged by the GP
- Permanently living at the nursing home
- Prescription of at least one antidepressant with one of the following Anatomical Therapeutic Chemical Classification (ATC) codes
  ○ Selective serotonin reuptake inhibitors (SSRIs): N06AB10 (Escitalopram); N06AB04 (Citalopram); N06AB08 (Fluvoxamine); N06AB03 (Fluoxetine); N06AB05 (Paroxetine); N06AB06 (Sertraline).
  ○ Serotonin-Norepinephrine Reuptake inhibitors (SNRIs): N06AX21 (Duloxetine); N06AX16 (Venlafaxine).
  ○ Tricyclic antidepressants (TCAs): N06AA09 (Amitriptyline); N06AA04 (Clomipramine); N06AA02 (Imipramine); N06AA10 (Nortriptyline); (Dosulepin); N06AA17
  ○ Noradrenergic and specific serotonergic antidepressants (NaSSAs) / Atypical antidepressants N06AX03 (Mianserin); N06AX11 (Mirtazapine).
  ○ Monoamine oxidase inhibitors (MAOIs): N06AF01 (Isocarboxazid);
  ○ Noradrenaline reuptake inhibitor (NARI): N06AX18 (Reboxetine).
  ○ Other antidepressant with effect on the serotonin-system: N06AX26 (Vortioxetine).
  ○ Melatonin agonists: N06AX22 (Agomelatine).

Patients are excluded if they meet the following exclusion criteria:

- Currently under treatment of a psychiatrist
- Participation in another similar ongoing trial
- Suspicion of a current major depressive episode, or suicidal ideations and behavior.
- Receiving end-of-life care
- Refusal to grant access to health information as part of the trial.

#### Identification and recruitment of patients

Using their electronic patient practice database, GPs assess the eligibility of all patients registered in their practice. GPs retrieve information on all potentially eligible patients. If more than 15 patients are eligible at one practice, the full list of potentially eligible patients is sent pseudo-anonymized to the research team by secured email. The research team then randomly selects a sample of 15 patients from each practice, using a computerized algorithm. The list with these 15 patients is returned to the GP.

#### Intervention and enhanced care-as-usual

##### ½ a day course

Before randomization, all participating GPs receive a ½-day course on the evaluation and treatment of neuropsychiatric symptoms that occur in patients with dementia, prescription of antidepressants to this population, and reasons to discontinue. Specialists in general practice, pharmacology, and non-pharmacological treatment will teach the course. The course is mandatory for the participating GPs but voluntary for the staff at the GP’s office. The course is preferably held with actual attendance but may be converted to an online meeting if the COVID-19 pandemic requires so. If a GP cannot attend, a summary of the course is given to the GP.

##### Visit at the GPs office by the research team

A member of the research team visits all included GPs after the course. At the visit, the trial allocation of the respective GP is disclosed and the GP is given the relevant material according to their allocation including the symptom assessment scale. The visit takes place before the first home visit of the GPs.

##### Symptom assessment scale

An email template containing 12 screening questions from the Neuropsychiatric Inventory Nursing Home Edition is given to the GPs to be distributed to the nursing homes before the home visit and one month after the home visit [32, 33].

#### Intervention group

At the instruction visit, the GPs in the intervention group receive the following three elements in addition:

1. Teaching material: The GPs receive a case-based teaching material to use at the nursing home. The material includes a description of BPSD symptoms, the mechanism, effect, and adverse events of antidepressants, and a rationale for deprescribing as well as non-pharmacological treatments of BPSD.
2. Pre-visit reflection tool: The GPs receive a checklist and an email template to ensure, that (1) the home visit is planned when members of the nursing home staff who know the patient best are at work and (2) the staff at the nursing home are instructed to contact the relevant relatives and inform them about the home visit.
3. Dialog tool: The dialog tool includes a list of questions to help the GP explore the nursing home staff’s, patients’, and relatives’ concerns and views on deprescribing antidepressants, as well as information on when to contact the GP.

The intervention has been developed during a tailoring process involving GPs, nursing home staff, interviews with patients, and other experts in the field. A more detailed description of the process is described in a separate article (unpublished data).

#### Control group

Patients attended by a GP allocated to the control arm will receive enhanced care as usual. GPs in the control group are not actively encouraged to withhold or reduce medication. However, since they attend our introductory course and are instructed to perform a medical chart review in the 10–15 included patients to evaluate neuropsychiatric symptoms, we expect them to consider and/or attempt reductions or discontinuations to a higher degree than outside of a trial setting. They do not receive any instructions or tools enhancing the involvement of nursing home staff or relatives. See Table 1 for an overview of what constitutes the intervention and control groups.

**Table 1:** List of what constitute the intervention and control group

Intervention group	Control group
- Educational session for GPs - Instructed to complete 10–15 home visits at the nursing home with optimizing antidepressants and other psychotropic medication - Instructed to evaluate neuropsychiatric symptoms before and after the visit using a structured form - Instructed to complete a teaching session at the nursing home with pre-defined teaching material - Instructed to contact the nursing home before the home visit to encourage the participation of regular staff and relatives in the home visit or to obtain information from regular staff and relatives before the home visit - Employment of a dialog tool	- Educational session for GPs - Instructed to complete 10–15 home visits at the nursing home with optimizing antidepressants and other psychotropic medication - Instructed to evaluate neuropsychiatric symptoms before and after the visit using a structured form

### Ensuring adherence

To improve adherence to the protocol, monthly emails will be sent to the participating GPs by the research team enquiring about any problems encountered concerning trial-related tasks. The emails include status on the home visits for the GP, a request for dates on already planned home visits, and a reminder to print a medication list for all included patients including a reminder to ensure follow-up data one month after each home visit. The GPs are encouraged to send an email when they have completed a home visit. No strategies for retaining patients have been applied since the patients are included as part of the GP cluster and could not opt out on their GP participating in a quality improvement study. However, patients and their relatives have to give informed consent for any diagnostic procedure or change of treatment according to the Danish Health Act. Furthermore, in Denmark, data for quality improvement studies can be collected without informed consent. Since the study is defined as a quality improvement study and informed consent from the patients is not required, it is possible to collect all relevant data as long as the GPs continue to be part of the study.

### Concomitant care

Any new interventions or initiatives introduced by local health authorities, municipalities, researchers, health organizations, and the like, aimed at improving healthcare for persons with dementia, will be recorded at the end of the study.

### Outcomes

#### Primary outcome

The primary outcome is any reduction of any antidepressant from pre- to post-intervention during the intervention period, in the intervention group compared to the control group, measured as a dichotomized response (reduction; yes/no). In the case of reduction of an antidepressant and an addition of another antidepressant, this will not be considered a reduction.

#### Secondary outcomes

- A proportional difference in the total number of antidepressants per patient prescribed from pre- to post-intervention
- A proportional difference of antidepressants from each class of antidepressants per patient prescribed from pre- to post-intervention
- A proportional difference of the number of antipsychotics, anxiolytics, hypnotics, anticonvulsants, analgesics, and anti-dementia medication prescribed per patient from pre- to post-intervention
- Change in outcome severity of behavioral and psychological symptoms scores as assessed by the Neuropsychiatric Inventory [32, 33] four weeks after the home visit
- Number of hospital admissions during the intervention period
- Number of falls requiring hospital admission or emergency department visit during the intervention period
- Mortality at the end of intervention (12 months after randomization)

#### Post-intervention analysis

- A proportional difference of the total amount of antidepressants per patient prescribed from randomization to end of study (at 12 months after randomization)
- A proportional difference of each class of antidepressants per patient prescribed from randomization to end of study (at 12 months after randomization)
- A proportional difference of the number of antipsychotics, anxiolytics, hypnotics, anticonvulsants, analgesics, and anti-dementia medication prescribed from randomization to end of study (at 12 months after randomization)
- Total number of hospital admissions at the end of study (at 12 months after randomization)
- Total number of falls requiring hospital admission or emergency department visit at the end of study (at 12 months after randomization)
- Mortality at the end of study (at 12 months after randomization)

### Sample size

The sample size is based on our primary outcome measure, any reduction of any antidepressant from pre- to post-intervention. We expect a mean reduction of 0.5 antidepressants per patient in the intervention arm, a change of 0.2 in the control arm, and an intra-cluster correlation coefficient of 0.10. We defined a *p*-value 0.05 as significant and deemed a power of 90% as sufficient. Based on the assumption of ten patients per general practice, a minimal sample size of 91 participants per arm is required, or 182 in total, corresponding to 18 practices in total. When considering a dropout among clusters of 20%, the total adjusted number of practices required amounts to 22.

### Allocation, sequence generation, and concealment

The study is a randomized controlled trial with the general practice as the unit of randomization. Allocation of the general practice is randomized using a computer algorithm. Allocation is 1:1 with intervention or control. The computer randomize allocation sequence will be concealed until all general practices are assigned. The research team will inform the participating GPs on their allocation.

### Blinding (masking)

The GPs are blinded to their allocation before recruiting patients. Due to the nature of the intervention, it will not be possible to blind the participants, providers of care, or other members of the research team after the allocation. The primary outcome is blinded to statistician and data manager is blinded until primary outcome is analyzed

## Data collection, management, and analysis

Table 2 shows the SPIRIT timeline of the study.

**Table 2:** SPIRIT timeline of study

	STUDY PERIOD			
	Enrollment	Allocation	Post-allocation pr. patient included	Close-out
TIMEPOINT	*−6 months*	*0*	*>0–3 months*	*>3–12 months*
ENROLLMENT OF THE GPs:				
Recruitment general practices	X			
Identification of patients	X			
Eligibility screen of patients	X			
Informed consent	X			
½ day course	X			
Allocation		X		
INTERVENTIONS FOR THE GP:				
Teaching material		X		
Pre-visit reflection tool		X		
Dialog tool		X		
INDIVIDUAL PARTICIPANTS				
Patients included in study	X			
Home visits including symptom scale			X	
Monthly medication charts printed	X		X	X
ASSESSMENTS:				
Monthly medication charts		X	X	X
Symptom scale			X	X
Hospital admission due to fall				X
Hospital admission any reason				X
Mortality				X

### Data collection before randomization

- General practice data: service number of general practice (Danish: *ydernummer*), address, name(s) of general practitioners, number of patients registered at practice, number of staff (including nursing staff and administrative staff), number of nursing homes for whom they are the primary physician. Information will be collected by the general practitioner and sent to the research team.
- Nursing home data: number of living spaces for residents, number of special living spaces for residents with dementia, owner status of the nursing home, protocols for the management of behavioral issues in residents, number of staff at the nursing home. Information will be collected by the research team.
- Patient demographics and medical history: social security number, when diagnosed with dementia, who prescribed the medication the first time and when was it prescribed, prior attempts to discontinue use, comorbidities, length of stay at the nursing home, the capability of providing informed consent to share health care data, former admittance to psychiatric wards after the age of 70, known depression diagnosis. Information will be registered on paper as a case report form (CRF) generated by the research team. The CRF is printed and data filled out by the GP. All data is collected manually by the research team.

### Data collection during the study period

**Fig. 1:**
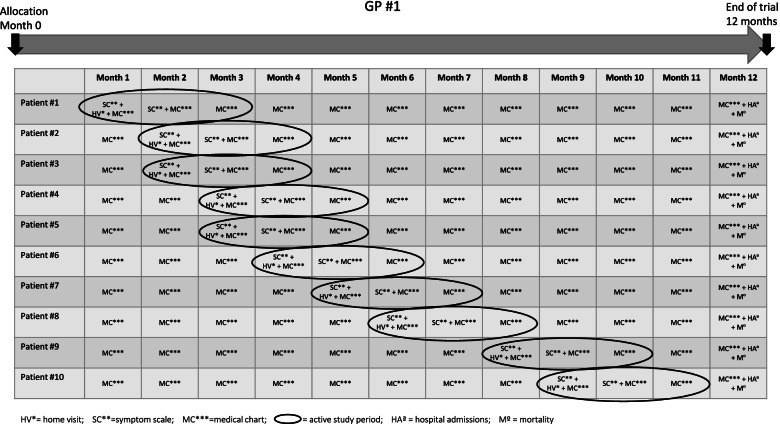
Explanatory example on how a GP conducts home visits and collects data. The figure is made as an explanatory example on how the patients from one GP may hold their home visits. For each GP, this may vary depending on how the visits are planned

The intervention period for each patient will comprise three months and should be completed before the end-of-study period of 12 months. Due to logistics, the 3 months intervention period will not start simultaneously for all included patients. Each GP will upon allocation have a full list of the 10–15 patients included in the trial in their practice. For each listed patient, the intervention period is defined as the home visit, follow-up with symptom scale and medical chart after 1 month, and medical chart after 2 months (Fig. 1). After completion of the 3-month intervention period for an individual participants, all data collected during the remainder of the (1-year trial) period, will be used for as post-intervention analyses. GPs are allowed to initiate the intervention in participants until 10 months after allocation.

A monthly medication chart will be collected by the general practitioner and sent to the research team.

A symptom assessment scale containing 12 items copied from the Neuropsychiatric Inventory will be collected by the nursing home staff and sent to the research staff by the general practitioner before the home visit and 3–4 weeks after the home visit.

The 12 items include yes/no screening questions in the domains of delusions, hallucinations, agitation, dysphoria, anxiety, apathy, irritability, euphoria, disinhibition, aberrant motor behavior, night-time behavior disturbances, and appetite and eating abnormalities.

### End-of-study data collection

Monthly medication chart, number of hospital admissions and mortality will be collected by the general practitioner from the electronic medical record and sent to the research team.

### Process evaluation

Observations will be carried out during the interventional study to access the feasibility and implementability of the intervention and to add to an explanation of factors that could promote or inhibit the uptake of the intervention among professionals involved in the trial. The evaluation will focus on identifying factors that promote and inhibit the core elements of the intervention:

1. How do the training courses and teaching material make sense to GPs and apply to daily work?
2. How does the pre-visit reflection tool integrate with the nursing home staff’s daily work and how does it add value to the dialog with the GP afterward?
3. How does nursing home staff and GPs interact with the dialog tool to facilitate shared decision making on optimization of BPSD

### Process evaluation data collection and analysis

To answer the three questions above, qualitative data focusing on interaction and collaboration will be collected by a researcher hired by the research team.

#### Observations

The training course for GPs will be video recorded to identify the professionals’ initial understanding and engagement in the trial. Observations will also be made in the intervention and during the home visits with reference to the integration of the novel components of the assessment process.

#### Interviews

During the implementation process, *semi-structured, face-to-face interviews* will be conducted with approximately 10 nursing home staff members, 10 GPs, and 10 relatives of participating patients. The interviews will explore how nursing home staff and GPs experience the core components of the intervention. An interview guide will be developed to ensure that the relevant theoretical dimensions are covered in the interviews. Furthermore, structured phone interviews will be conducted with a sample of 10 relatives focusing on their experiences with the professional’s attempted effort in including them as relatives and the intensified cross-professional dialog about the patient.

### Interpretation of observational data and interview data

Qualitative data will be coded and summarized systematically by charting according to key themes of implementation, mechanisms, and context. The analysis will address the objectives set out above, and emerging hypotheses tested according to all the relevant data. We will use normalization process theory to assess the extent to which the new processes become embedded in daily practice [34].

#### Data management

The trial starts when approval by the relevant Danish authorities has been granted. The study adheres to all Danish laws governing medical research. The General Data Protection Regulation is upheld, and data is stored and handled according to this. The study owner is responsible for upholding laws and ensuring the confidentiality of data. The research team collects and subjects’ data to double entry in Access® at a secure server. All data that can identify participants will be encrypted and stored securely on password-protected servers with continuous transaction logging at servers on the University of Copenhagen. Data is accessed by the research team through the secure server. Trial data is stored in accordance with the data policy of the University of Copenhagen. Data is saved for 5 years after data collection and will hereafter be anonymized or terminated.

#### Statistical methods

Binary outcomes will be analyzed using a logistic regression model with generalized estimating equation (GEE) models to take into account clustering and multiple measurements. Continuous variables will be analyzed using mixed models to take into account clustering and multiple measurements. Missing data is analyzed by multiple imputation. A statistical analysis plan will be generated before the allocation will be disclosed.

## Monitoring

### Data monitoring

Due to the limited duration of the study and the consideration that the intervention carries minimal risk, there will be no need for a formal data monitoring commission, auditing, or interim analysis. Furthermore, GPs may reinitiate treatment with antidepressants if medically indicated.

The study has been approved by the University of Copenhagen Data Protection Agency (Case no.: 514-0562/20-3000). According to Danish law, there is no need to apply for the approval of the National Danish Data Protection Agency when regional approval has been given.

A data process agreement with each GP will be attained and signed before the collection of data.

### Harms

A potentially harmful effect of discontinuation is withdrawal symptoms. To minimize this risk, participating GPs will receive information on the proper and safe discontinuation of antidepressants. Discontinuation of antidepressants may hypothetically induce an exacerbation of the behavioral and psychological symptoms of dementia. Given the current body of evidence demonstrating the minimal or absent effect of antidepressants in BDPS, we deem this risk to be low. Contrarily, it has been shown that antidepressants are associated with an increased risk of falls in the older population.

Our outcome measures include some potential adverse effects of antidepressants, which allows us to closely monitor the potentially negative effects of our intervention. No additional adverse events are systematically registered as part of the study, but since the patients are living at nursing homes with 24-h staff, any worsening can be discovered and reported to the GP.

Moreover, GPs are not precluded to reinitiate treatment with antidepressants if medically indicated.

## Ethics and dissemination

### Research ethics approval

The study has been submitted to the Research Ethics Committee of the Capital Region of Denmark. As defined by the Danish Act on Research Ethics of research projects section 2, the project does not constitute a health research project but considers a quality development project. Therefore, the current study does not require approval from the Committees on health research ethics of the Capital Region of Denmark (Journal no: H-20084023).

Furthermore, the Danish Patient Safety Authority also considers it a quality development project (journal no: R-20079697), and they have waivered the need for approval.

### Protocol amendments

The trial will be conducted in accordance with the protocol. If any modification is required due to possible benefits for or safety issues of the patients, it will require a formal amendment. A formal amendment requires the approval of the Ethics Committee before implementation. Minor corrections that are seen as administrative rectifications to the protocol and have no effect on the conduction of the trial will be agreed upon by the research group and will be documented in a memorandum.

### Informed consent

Due to the cluster-randomized design, patients cannot give individual informed consent to participation, since all patients listed to participating GPs will be somehow subjected to the change of procedures in the practice. The intervention consists primarily of a dialog tool, and the GPs are encouraged to follow the Danish national guidelines regarding optimization of pharmacological treatment; however, they are not obliged to discontinue or change medication and patients are not obliged to consent to any change in medication.

Danish law requires written informed consent for passing on health information, but it is allowed to pass on health information for quality assurance projects without informed consent from patients [35]. Since the quality assurance aspect of this study requires access to patient data from all involved patients in order to evaluate the quality of the medical chart reviews in both groups, it is allowed to pass on relevant health information without informed consent.

### Confidentiality

All data will be stored securely according to the guidelines set forth by the General Data Protection Regulation. All reports, data collection, process, and administrative forms will be identified by an identification number only to maintain participant confidentiality. All records that contain names or other personal identifiers will be stored separately from study records identified by a code number on a secure server. All local databases will be secured with password-protected access systems. Participants’ study information will not be released outside of the study without the written permission of the participant.

### Declaration of interests

There is no conflict of interest

### Access to data

The research team will have access to all data.

### Ancillary and post-trial care

Not applicable. No additional care is provided in the trial.

### Dissemination policy

The results from the study will be published in peer-reviewed journals. The final list and order of authors follow the contribution from each researcher and follows the Vancouver rules and the guidelines from The Danish Committees on Scientific Dishonesty.

## Discussion

This cluster-randomized controlled study aims to test the effectiveness and feasibility of an intervention that encourages discontinuation of antidepressants and other psychotropic medication in institutionalized older people with dementia. The effectiveness is assessed in terms of reduction in the number of antidepressants.

Possible shortcomings of this cluster randomization are that in both the control and the intervention group participants receive education on optimizing the treatment with a focus on discontinuation of antidepressants. Furthermore, the enhanced care as usual group is requested to make an additional visit to the patients, enhancing the change of a planned reduction that would not otherwise have been attempted. This might diminish the observed efficacy of the intervention.

A possible limitation in the study is the choice to exclude patients currently under treatment by a psychiatrist. Excluding patients under treatment of a psychiatrist could result in a preferential selection of mild and moderate forms of BPSD in our study, which to a certain extent compromises the external, though not internal validity of our results. However, the intervention is intended for nursing home GPs and not psychiatrists. We have interviewed GPs during the development of the intervention, and they expressed reluctance to change psychotropic medication, if the patient was under (current) treatment by a psychiatrist. Moreover, a psychiatrist would scrutinize the indication for the individual psychotropic medications and thereby already optimize the treatment. Including these patients would therefore lead to an underestimation of the effect of the intervention.

Although we regard an intervention period of 3 months sufficient to assess the feasibility of the intervention concerning reduction, it might be too short to detect the effect of discontinuation of antidepressants in terms of mortality, morbidity, and falls.

We cannot fully preclude the risk of post-allocation bias. GPs were instructed to recruit patients before allocation, thereby mitigating preferential selection of patients. However, after allocation, the physician may apply the intervention first to the mild or uncomplicated patients and may not be able or inclined to apply it to the severely ill or complicated patients. This risk may be unbalanced between the two arms, since the incentive to pick mild or uncomplicated patients may be stronger among the physicians in the intervention group, as they must invest more time and effort in delivering the intervention compared to the care-as-usual group, who only have to report on the severity of dementia symptoms occasionally. However, as the physicians receive reimbursement per patient, this might encourage physicians to deliver the intervention to all assigned patients.

Research in a vulnerable patient population, for instance, people with dementia inevitably raises ethical concerns. Many of these patients have limited or no capacity to give informed consent and often lack a legal guardian who may provide a substitute decision. The evidence base for therapeutic options for patients with dementia is therefore scarce and therefore makes a study such as this even more important.

## Trial status

Protocol version 1, 29.09.2021.

Initiation of recruitment of patients by October 2021. Expected to be completed by November 2022.

## Supplementary Information

**Additional file 1.** SPIRIT Checklist.

## Data Availability

The research team will have full access to all data.

## Abbreviations

BPSD: Behavioral and psychological symptoms in dementia
GP: General practitioner
